# Case Report: The woman with the big heart—an imaging-guided attempt of surgical reduction

**DOI:** 10.3389/fcvm.2024.1263905

**Published:** 2024-01-26

**Authors:** Katharina Huenges, Patrick Langguth, Christina Grothusen, Grischa Hoffmann, Julia Kapahnke, Assad Haneya, Jörg Strotmann, Jochen Cremer

**Affiliations:** ^1^ Department of Cardiovascular Surgery, UKSH Kiel, Kiel, Germany; ^2^ Department of Radiology and Neuroradiology, UKSH Kiel, Kiel, Germany; ^3^ Internal Medicine I, St. Johannes Hospital Dortmund, Dortmund, Germany; ^4^ Department of Cardiology, Städtisches Krankenhaus Kiel, Kiel, Germany

**Keywords:** mitral, mitral valve, atrial, cardiac surgery, imaging

## Abstract

In a female patient with acute cardiac decompensation, an auxiliary finding of a giant left atrium emerged. The surgical therapy of the atrial reduction, in addition to a mitral valve replacement and a coronary artery bypass grafting, is hereby presented.

## Introduction

Atrial reduction surgery is not commonly performed in the daily cardiac surgery routine. There have been only very scarce data of cases and techniques published so far (1–3). In this patient, the preoperative imaging provided a safe surgical planning, and a relevant reduction of the atrial size was possible.

## Case description

**Figure 1:**
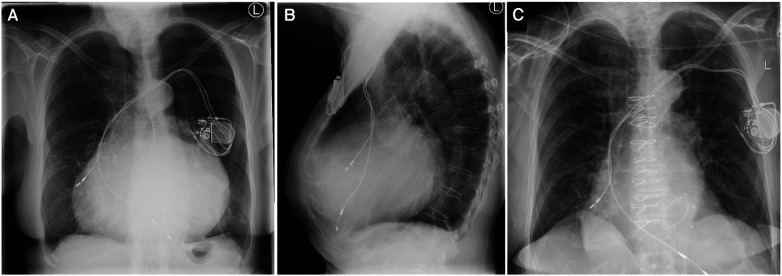
(**A**) Chest x-ray prior to surgery, (**B**) lateral chest x-ray prior to surgery, and (**C**) chest x-ray after surgery.

**Figure 2:**
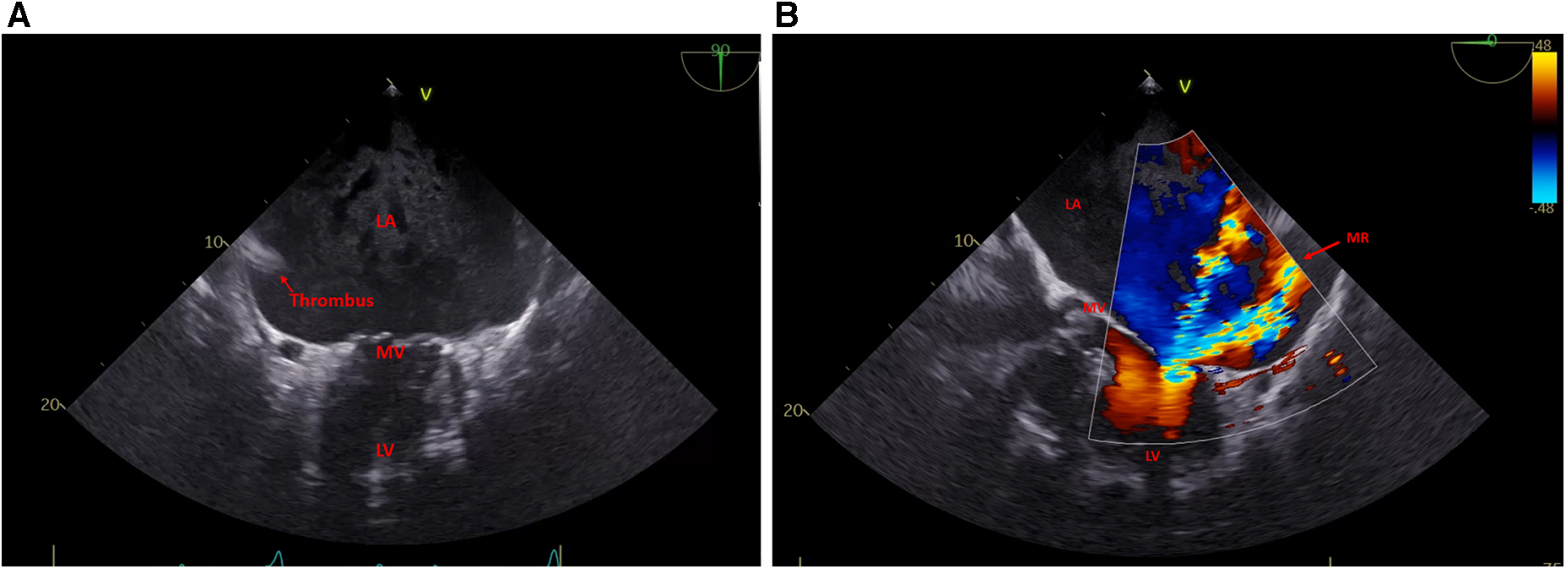
(**A**) Left atrium (LA) with thrombus formation two-chamber view and (**B**) mitral regurgitation.

A 72-year-old female patient with a giant left atrium was presented to our department. Permanent atrial fibrillation has been known for the past 13 years. Due to severe cardiac decompensation, the patient had to be admitted to an external cardiology clinic. The chest x-ray at the admission date showed a severe mediastinal enlargement (Figures 1A,B). Echocardiography revealed a massive biatrial dilatation, with a pronounced dilatation of the left atrium and intra-atrial thrombus masses were detectable. The left ventricle (LV) function was normal. Transthoracic and transesophageal echocardiography were not able to reliable measure the atrial dimensions possibly due to beam width limitations. Further examinations revealed a severe mitral regurgitation with secondary pulmonary hypertension, a moderate tricuspid regurgitation, and a two-vessel coronary artery disease. A significant left anterior descending (LAD) stenosis was treated interventionally with a drug eluting stent (DES) implantation, but a moderate circumflex artery stenosis remained untreated. The decision for the LAD intervention was made by the external cardiologist due to the decompensated condition of the patient. Anticoagulation was performed with edoxaban, but still, left atrial thrombus formation was detectable (Figure 2A, Supplementary Video S1). The mitral regurgitation was due to degeneration of the anterior and posterior leaflets (Carpentier IIIa, Figure 2B), and an additional annular calcification was notable. Optimized heart failure medication was given, and after careful recompensation, the patient was transferred to our department for surgical therapy. Anticoagulation therapy was switched to therapeutic low-molecular-weight heparin.

**Figure 3:**
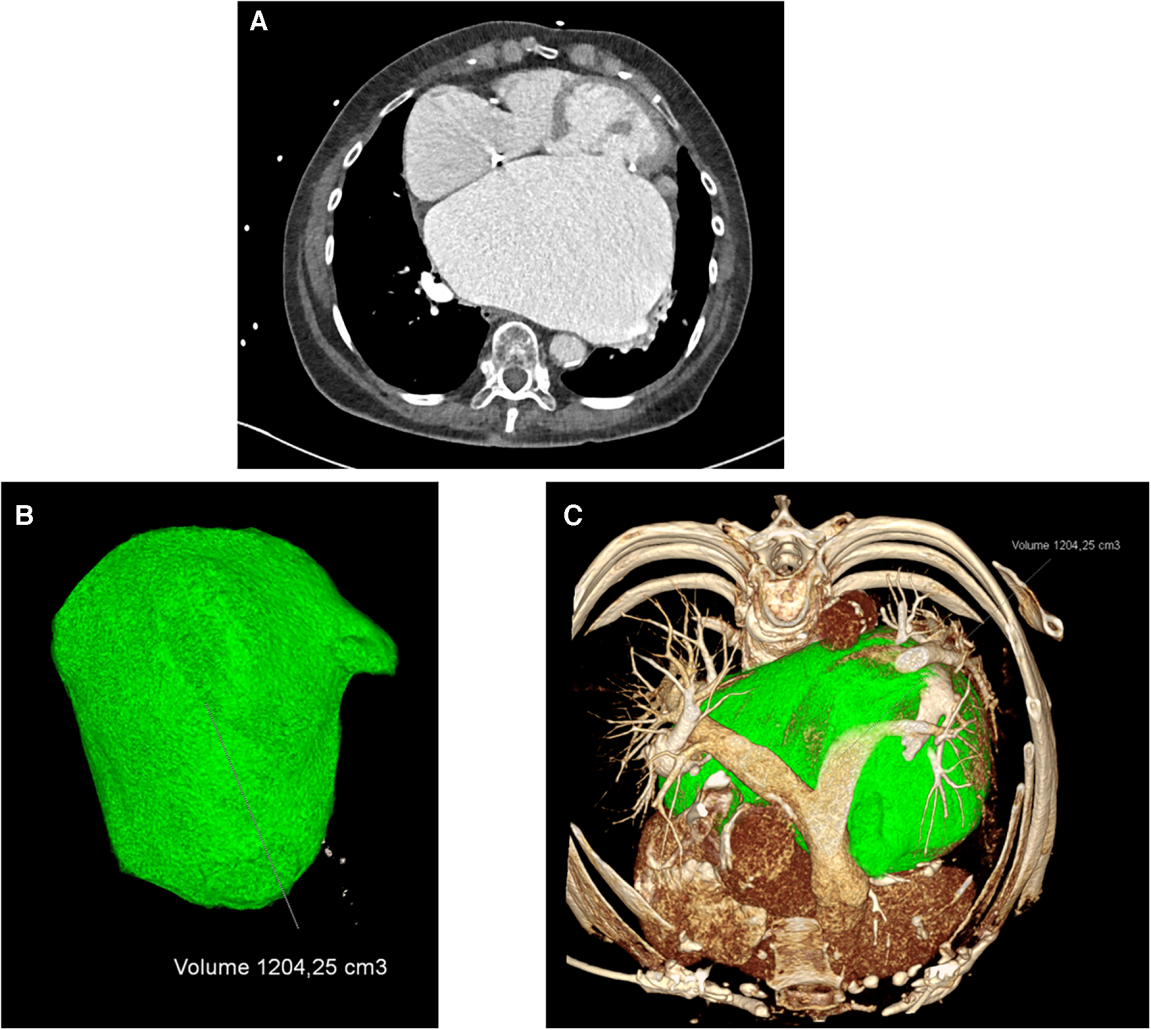
(**A**) CT scan prior surgery, (**B**) left atrial 3D volumetric measurements, and (**C**) left atrial (green) intrathoracic 3D dimensions.

Computed tomography (CT) with additional CT angiography was performed for better understanding of the cardiac structures. With the CT scan, the left atrial dimensions were measurable, the axial diameter was 12.5 cm × 15 cm, and the volumetric calculation showed 1,204 ml (Syngo.via VB60A, Siemens Healthineers, Erlangen, Germany) (Figure 3).

## Case report timeline


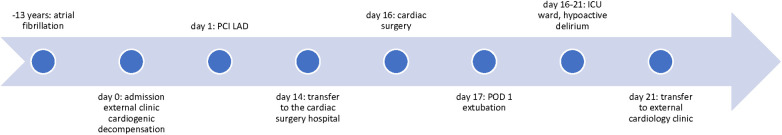


## Surgical technique

Cardiac surgery was performed in standard fashion with general anesthesia, using median sternotomy as the approach. For the heart–lung machine, bicaval venous cannulation and aortic direct cannulation were applied. In moderate aortic atherosclerosis, cautious aortic cannulation and cross-clamping were necessary. Antegrade and retrograde blood cardioplegia were used as it is our standard cardioplegic approach.

The left atrium was opened via the interatrial sulcus. For improved vision, a retractor was inserted. The distance between the left and right pulmonary veins was over 10 cm. Close to the left atrial appendage, a 2 cm × 1.5 cm × 1.5 cm white thrombus was found and extracted. For reduction of the atrium, a longitudinal incision was made between the area of the coronary sinus until the middle line between the pulmonary veins and a tissue reduction of 3–4 cm was performed and with a 4-0 Prolene suture using the double-running technique. A second oblique oval-shaped excision of 6 cm × 7 cm of tissue parallel to the atrioventricular grove was performed with the same suturing. With these two incisions and steps of tissue removal, a relevant reduction of the atrial dimension was possible. The surgery was continued by aortocoronary bypass grafting of the first marginal branch of the circumflex coronary artery using a venous graft. It was followed by a mitral biologic valve replacement (33 mm Hancock II, Medtronic) as reconstruction of the native mitral valve was not feasible due to severe annular sclerosis.

Under catecholamine and inotropic support, the patient was transferred to our intensive care unit. After careful weaning, extubation was possible on the first postoperative day. Due to a slight hypoactive delirium, ICU observation was necessary for the first 5 days, and in a stable hemodynamic situation, the patient was then transferred to the external cardiology department for further treatment.

Echocardiography revealed a competent mitral valve prosthesis without any paravalvular leakage, the left atrium seemed to be reduced, but still a reliable measurement was not possible.

A follow-up computed tomography was performed 2 weeks after the surgery due to still slightly impaired renal function, this time only as a native CT scan without contrast agent. The size of the left atrium was reduced optically, and careful estimation showed a 30%–40% reduction of the atrial size due to surgery. However, after the radiological analysis and reconstruction, the left atrial reduction was approximately over 50% of the atrial initial size (volume before: 1,205 ml; volume after: 582 ml).

After an initial very promising course after the surgery, the patient developed COVID-19 significantly 3 months after the surgery and had to be rehospitalized, but from the cardiac aspects, the patient showed no complications.

## Discussion

Atrial reduction surgery is not commonly performed in the daily cardiac surgery routine. There have been only very scarce data of cases and techniques published so far (1–3). The main reason is that even if mitral valve regurgitation or tricuspid valve regurgitation is severe and ongoing for many years, it is not invariably leading to giant atria. After correction of the heart valve pathology, until a certain degree, an atrial re-remodeling can occur with an autonomous reduction of the atrial size.

In our patient, we did see a giant left atrium, and even after the mitral valve surgery, remodeling with a significant atrial reduction was not very likely. Since intra-atrial thrombus formation had already occurred despite anticoagulation therapy, we did see the need for an attempt of atrial reduction. With the CT reconstruction, a safe operative plan for the surgery was possible. The reduction technique applied in this case led to a relevant decrease in left atrial size. The choice of the tissue reduction line has the potential for a safe reduction without changing the native normal atrial configuration. Sparing the superior atrial wall (compared to the plication techniques) further reduces the risk of permanent atrioventricular node disorders.

Informed consent was obtained from the patient included in this study.

## Data Availability

The original contributions presented in the study are included in the article/**Supplementary Material**, further inquiries can be directed to the corresponding author.
